# Characterization of Indicators for Adaptive Human-Swarm Teaming

**DOI:** 10.3389/frobt.2022.745958

**Published:** 2022-02-17

**Authors:** Aya Hussein, Leo Ghignone, Tung Nguyen, Nima Salimi, Hung Nguyen, Min Wang, Hussein A. Abbass

**Affiliations:** School of Engineering and Information Technology, University of New South Wales, Canberra, ACT, Australia

**Keywords:** adaptive autonomy, human-swarm interaction, mission performance indicators, interaction indicators, complexity indicators, automation indicators, human cognitive state assessment

## Abstract

Swarm systems consist of large numbers of agents that collaborate autonomously. With an appropriate level of human control, swarm systems could be applied in a variety of contexts ranging from urban search and rescue situations to cyber defence. However, the successful deployment of the swarm in such applications is conditioned by the effective coupling between human and swarm. While adaptive autonomy promises to provide enhanced performance in human-machine interaction, distinct factors must be considered for its implementation within human-swarm interaction. This paper reviews the multidisciplinary literature on different aspects contributing to the facilitation of adaptive autonomy in human-swarm interaction. Specifically, five aspects that are necessary for an adaptive agent to operate properly are considered and discussed, including mission objectives, interaction, mission complexity, automation levels, and human states. We distill the corresponding indicators in each of the five aspects, and propose a framework, named MICAH (i.e., Mission-Interaction-Complexity-Automation-Human), which maps the primitive state indicators needed for adaptive human-swarm teaming.

## 1 Introduction

Recent technological advancements have enabled the realization of swarm systems that can include large numbers of robots. Using local communication and distributed coordination, these robots can achieve complex global behaviors that can be utilized in a wide range of applications. However, fully autonomous swarms that are free from human supervision are hard to realize due to technological and ethical impediments. Technologically, although artificial intelligence could surpass human intelligence in a number of applications, it is not expected to outperform general human intelligence in the near future (Fjelland, 2020); the success of fully autonomous swarm operations in dynamic and complex environments and in the absence of human oversight remains a vision for a reasonably distant future. Ethically, full autonomy can be undesirable due to the responsibility and accountability issues (Nothwang et al., 2016; Wachter et al., 2017). Therefore, the use of a human-in-the-loop model is still an important bridge to ensure the safety of operations, especially for critical and sensitive applications in medicine and military (O’Sullivan et al., 2019; de Ágreda, 2020; Verdiesen et al., 2021). Human-swarm systems will remain the most feasible path, at least for the foreseeable future, for adopting swarm systems in real environments.

In human-swarm interaction (HSI), humans and swarms need to act as a team to optimize common mission objectives. The human and the swarm are assigned complementary roles with the aim of combining their skills efficiently and in a manner that achieves mission goals. Generally, there are three types of autonomy in HSI, namely, the fixed autonomy, human-based flexible autonomy, and agent-based adaptive autonomy. Fixing the level of autonomy within the swarm produces a rigid system in which the human can experience undesirable workload levels. This situation applies regardless of the level of autonomy that the swarm exhibits. If the level of autonomy is low, the human carries most of the load, with the end result of overloading the human. In contrast, if the swarm’s level of autonomy is high, the human could become underloaded. Both situations are undesirable as they can lead to difficulties in sustaining human situational awareness (SA) and to decreases in human performance (Endsley and Jones, 1997) and engagement level (Endsley, 2017). In the long term, fixed autonomy has been criticized for the associated skill degradation (Hilburn, 2017).

To realize effective interaction, humans and the swarm need to coordinate their actions throughout the mission to maintain acceptable levels of workload while ensuring that the tasks are performed effectively. This coordination can be assigned to the human or to a coordinating agent. Designating the human for the coordination can be time-consuming and potentially unsafe in situations where the human operator is naturally overloaded because of task demands (Chen and Barnes, 2014). In addition, task delegation to a human’s hands is subjective and depends on human factors that reach beyond the realm of workload (e.g., emotional stress). Some operators would place more constraints on automation at the expense of time, while others might place fewer constraints on the automation at the risk of the automation’s behavior possibly diverging from the operator’s intent (Miller et al., 2005).

To overcome the aforementioned challenges, the coordination can be performed by an adaptive agent at the interface between the human and the swarm to facilitate the interaction by monitoring and managing the states of different entities in the system. The agent decides whether the level of autonomy should be increased or decreased based on the state of the mission as assessed by certain input indicators. The accurate assessment of the mission is important to avoid sudden changes that can be inappropriate or annoying for the human (Chen and Barnes, 2014). Effective and efficient HSI calls for intelligent agents capable of adaptively and dynamically allocating the functions required to perform a given mission between humans and the swarm.

Adaptive autonomy (Abbass et al., 2014b; Hentout et al., 2019; Onnasch and Roesler, 2020; Tanevska et al., 2020) has been attracting increasing interest in the literature of human-automation interaction (HAI) and human-robot interaction (HRI) as a flexible autonomy scheme that acknowledges the dynamic and uncertain nature of the interaction. In adaptive autonomy, the functions required to achieve a mission are identified in advance. For example, if the mission is to drive a vehicle from its current location to a goal, the functions to achieve this mission could include the following: 1) an environment monitoring function, 2) a current car-state estimation function, 3) a hazard detection function, 4) a route planning function, 5) a vehicle dynamic function, and 6) a vehicle steering function.

An artificial intelligence (AI) agent is responsible for the adaptive control that dynamically allocates these functions to the human and the autonomous vehicle(s) based on the current requirements of the task and the states and capabilities of its potential performers (humans and machines). Adaptive autonomy has demonstrated its ability to enhance the performance of the overall human-machine interaction and mission (Chen and Barnes, 2014). This enhancement is attributed to its ability to reconcile conflicting requirements within the interaction (e.g., to make best use of the automation while ensuring that the human does not lose situational awareness or his/her level of engagement). The function of adaptive autonomy can be described by two questions: when and how. The when question is concerned with evaluating the current state of the overall system-of-systems to determine whether an adaptation is needed. The how question is concerned with generating new task assignments and corresponding user interface changes. Such a requirement comes with a few challenges that include determining how to dynamically adjust the level of autonomy of different players, how to strengthen mutual trust, and which mechanisms are required to facilitate situational awareness (SA) of the players (Humann and Pollard, 2019; Cavanah et al., 2020). The adaptive AI agent needs to form its own contextual awareness in order to be able to decide when adaptation is needed. Such contextual awareness requires continuous assessment of the states of different components in the overall system. Therefore, the main focus of the paper is placed on the indicators that will enable the AI agent to answer the when question.

In a taxonomy of triggers for adaptive autonomy, Feigh et al. (2012) proposed five categories: operator, system, environment, task/mission, and spatio-temporal triggers. Their work offered a high-level overview of the adaptive agent without delving into the indicators required for each type of trigger. Moreover, a swarm context is a decentralized context; designing the adaptive agent for decentralized applications is a research area that is in its infancy. We will fill this gap by distilling from the literature the list of indicators that could provide state assessment of different components in HSI. The aim of this paper is to identify and fuse together different categories of indicators needed for adaptive autonomy in HSI, and to study how the state of each component can be quantified using synthesized indicators from the literature. Five categories of indicators are identified for adaptive autonomy in HSI; three of these categories (mission complexity, human state indicators, and mission performance) have been used in adaptive human-machine interaction, while the other two categories (interaction indicators and swarm automation indicators) have been used in studies for HSI. Due to the enormous literature in each of these categories, this paper reviews the featured literature and summarizes only representative studies to show different ways to operationalize each category of indicators.

In Section 2, we present a framework for adaptive autonomy that facilitates the realization of effective HSI. In Section 3, we distill five groups of indicators that are necessary for an adaptive agent to operate properly. These five classes of indicators are then discussed in detail in Sections 4–8. In Section 9, we present the MICAH framework that combines the five types of indicators, and illustrate an walkthrough example with discussions on the working scenarios, followed by conclusion and future work in Section 10.

## 2 Framework for Adaptive Autonomy in Human-Swarm Interaction

Despite the increasing interests in deploying swarms in real-life applications, humans will remain a critical part of the autonomous control loop due to legal/ethical concerns and practical considerations, at least for the foreseeable future (Nothwang et al., 2016). A framework for adaptive autonomy in HSI brings together both human and swarm agents to optimize the performance of the overall system. The framework aims to achieve seamless and adaptive interaction between humans and the swarm to maximize mission objectives. To accomplish this goal, a significant number of state indicators is needed for the accurate evaluation of the state of different components in the overall system.

Conventional adaptive systems trigger adaptation processes in specific situations or for particular tasks (Feigh et al., 2012). These systems resume the default setting once the trigger is no longer active. However, as the situation and context evolve, the once-adequate adaptation strategy applied by conventional adaptive systems may become inadequate (Fuchs and Schwarz, 2017). To address this problem, dynamic adaptations based on a diverse set of indicators are required. However, these indicators are normally spread across different fields. For example, to assess the human mental states, the indicators would come from cognitive psychology, behavioral psychology and human factors (Debie et al., 2019). Meanwhile, to assess the success of an autonomous decision, the indicators would come from system engineering, control theory, and AI.

**FIGURE 1:**
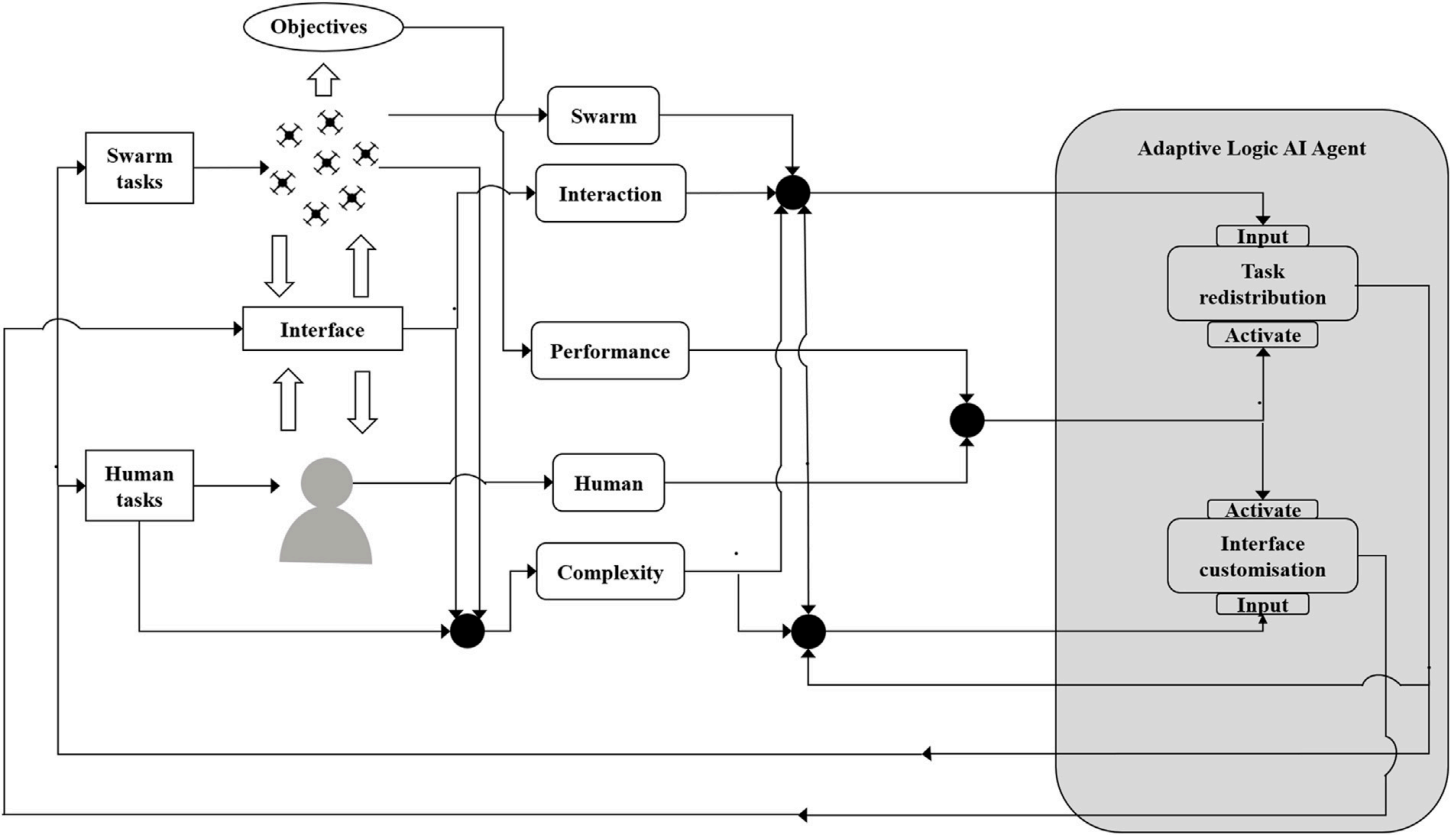
Framework for adaptive autonomy in HSI.

In this paper, we synthesize from this wide interdisciplinary literature those groups of indicators required to provide state assessment for the adaptive AI agent. A conceptual diagram of the framework is presented in Figure 1 for the adaptive AI agent.

The adaptation strategy of the adaptive AI agent can be viewed as two high-level steps: a monitoring and state assessment step followed by an adaptation step. The adaptive agent could take many forms, including a rule-based system, neural network, or other forms of symbolic and non-symbolic approaches. Below is an example of how the indicators could support the adaptive AI agent based on real implementations from our previous work (Abbass et al., 2014a; Abbass et al., 2014b; Harvey et al., 2018).

Psycho-physiological sensors collect the cognitive information of the human operator, which is then transformed into a series of human state indicators, including workload, fatigue and focused attention indicators (Debie et al., 2019). Integrated with information of the current task and system states, these human state indicators are used by the adaptive AI agent to decide whether to adapt or not. For example, high workload and fatigue may compromise the performance of the human operator, so the system could increase the autonomy level of the swarm or a subgroup of the swarm to allow the human operator to only focus on the most critical task. In another case, the lack of cognitive attention of a human may cause serious consequences, especially in emergency situations. Therefore, the system could lower the autonomy level of the swarm or a subgroup of the swarm and update interaction modes and visualization to maintain the human states within a safe range.

## 3 Indicators for Adaptive Human-Swarm Interaction Systems

In this section, we will first use a scenario to explain adaptive autonomy in an HSI context and then discuss the components and requirements for a good set of indicators.

### 3.1 Human-Swarm Scenario

Consider a search-and-rescue (SAR) scenario where victims are spatially spread in an urban area after an earthquake. An uninhabited aerial vehicle (UAV) remotely operated by a human pilot is used to guide a swarm of uninhabited ground vehicles (UGVs). Each UGV has a camera and a laser imaging, detection and ranging (LIDAR) sensor. The sensors on the UGVs are used to detect victims and to retrieve and move them to drop-off/collection locations in order to offer them first aid services before transporting them to a hospital. The mission objective is to maximize the number of victims retrieved within the allowed time frame.

The victims may move to other locations as they search for rescuers or appear and disappear at any time due to noise in the sensors. The human UAV operator has better sensing technologies in his/her UAV and is thus better able to assess the presence or absence of victims in an area. However, the nature of the UAV does not allow it to access areas that the UGVs can access. The swarm of UGVs is able to more closely approach the victims in order to better identify them. The swarm will always be attracted to the closest victim, but the human may be able to suggest better routes by considering the general disposition of victims and their future movements. Nevertheless, latency in the communication between the UAV and UGVs could cause delays that reduce the worthiness of information. Human control of the system, therefore, could lead to sub-optimal behavior when over-use causes network overload, and an increase in human workload could result in an increase in human error.

In summary, high levels of autonomy throughout the mission are undesirable because the swarm can make errors in the identification of victims and may act on biased local information alone. At the same time, low levels of autonomy will increase human workload, causing an increase in human errors and an increased network load. The role of adaptive autonomy in this scenario is to balance the load on the human and the autonomous system to ensure that the overall system of systems acts efficiently and effectively.

### 3.2 Different Requirements

Different components within the interaction can have unique requirements for autonomy adaptation. To begin with, the success of the mission is the ultimate goal of the interaction and the primary purpose for forming a team of humans and a swarm. Therefore, mission performance indicators are crucial to ensure a successful mission. In the scenario above, a high rate of collection is a signal that the current distribution of responsibilities between the human operators and the swarm of UGVs is appropriate. On the other hand, a low rate of victim collection is a signal that some improvement is necessary, but it does not provide, on its own, sufficient information regarding the source of the problem, that is, whether the problem lies in the swarm, the human, or their interaction.

Swarm automation indicators could offer information on how well the swarm is performing. For instance, if the swarm is found to be in a chaotic state, breaking apart or experiencing a high number of collisions, it is an indication that the swarm is one of the contributors to the low performance. However, whether more or less human intervention is needed to return the swarm to a stable state is a question for which the answer requires information from the interaction indicators.

Indicators of the effectiveness of the interaction between human and swarm provide information regarding whether the increase in human involvement in one task causes an increase in the rate of victim collection. If so, then giving less autonomy to the swarm might be considered to improve the results. However, such a setting can overwhelm the human. Therefore, we need to keep an eye on the human cognitive states to ensure that they remain within the desirable range. Otherwise, if the human continues to be overloaded for a prolonged amount of time, the performance of the team risks degradation unless some amount of work is lifted from the human. However, which task to offload from the human is a non-trivial question. This decision has to be based on an understanding of how each task is contributing to human workload, as well as the potential effects of different task assignments on the experienced workload. Fortunately, this information can be obtained from the analysis of task complexity using task complexity indicators.

Following this discussion, we contend that these five categories or components (mission performance, swarm automation, interaction, human cognitive states, and task complexity) are of crucial importance to the adaptive agent. Thus, the corresponding classes of indicators are considered for state assessment within our framework. In the next sections, we discuss each of these classes of indicators.

## 4 Mission Performance

Automation equips an automaton with functions to process and/or execute tasks. The level of automation, therefore, represents an agent’s capacity to perform a task, while autonomy expresses “the freedom to make decisions” (Abbass et al., 2016) afforded by the opportunity that exists to allow an agent to act. Autonomy carries negative risks when the capacity of an agent, that is, automation, is conceptually less than the capacity required to perform a task given an opportunity within a mission.

The primary aim of the team composed of the human and the swarm is to perform the mission successfully, which calls for indicators to allow the team to monitor progress towards the mission’s objective(s) in order to take corrective actions and/or adapt accordingly. We distinguish between how to measure effectiveness (achieving mission success) and efficiency (achieving the success using minimal resources/time) of the system performance in HSI. While mission effectiveness indicators are success indicators for the HSI in achieving mission objectives, mission efficiency is more about how competent the human and the swarm are in achieving these objectives. Details of mission effectiveness and efficiency metrics are discussed in the following subsections.

### 4.1 Mission Effectiveness

Mission effectiveness is considered as a factor of assessing how well an HSI achieves the mission. Olsen and Goodrich (Olsen and Goodrich, 2003) emphasize the importance of verifying mission effectiveness in designing an appropriate model of HSI. In the literature, various metrics are chosen to measure mission effectiveness. Generally, the overall goal is to maximise mission effectiveness, but the exact measurements chosen would depend on the nature of the mission. Below, we give examples of metrics proposed in the literature to measure the effectiveness of typical HSI missions, namely, SAR and navigation.

Under the umbrella of the National Institute of Standards and Technology (NIST), Jacoff et al. (2001a) and Jacoff et al. (2001b) proposed a list of quantitative and qualitative metrics to evaluate the performance of a team of humans and a group of autonomous ground vehicles in an SAR mission. These metrics were the number of localized victims, the number of found obstacles, the number of packages supplied to victims (such as first aid kits, radios, or food and water), and quality of communication with humans. These metrics add reward points to the overall measure of mission effectiveness. Points are lost when the team causes damage to the surrounding environment, victims, or themselves. The three primary metrics used for effectiveness were as follows: recovery rate, representing percentage of victims localized vs. number within the debris; accuracy rate, the percentage of the number of correctly localized victims from the total number of localized victims; and the total damage relating to victims and the environment.

In navigation missions, Steinfeld et al. (2006) introduced five key measures of effectiveness: percentage of navigation tasks successfully completed, area coverage, deviation from a planned route, obstacles that were successfully avoided, and obstacles that were not avoided but could be overcome.

Focusing on SAR missions with a time-critical requirement, Crandall and Cummings (Crandall and Cummings, 2007) conducted an HSI scenario where mission effectiveness was evaluated through two measurements: the number of objects collected (OC) and the number of robots remaining (RR) at the end of the mission.

Chien et al. (2012) proposed a list of measurements used to evaluate the effectiveness of the interaction between humans and a group of autonomous robots. These measurements are the number of victims rescued, the distance travelled and the percentage of area covered.

Harriott et al. (2014) introduced metrics to monitor the progress of a mission: area coverage and mode error or total damage. They also proposed a metric for resource collection to represent the progress of a foraging mission. They used Eq. 1 to measure resource collection as follows:

$${\text{S}}_{\text{i}}\left(\text{t}+1\right)={\text{S}}_{\text{i}}\left(\text{t}\right)-\text{N}\times \text{e}$$ (1)

where a resource (*i*) with size (*S*) is collected through time steps (*t*). *e* > 0 represents the amount of resources to be reduced by an agent, and *N* is the number of agents within *r*_*e*_ metres of the location of the resource. Low values for *S*_*i*_(*t* + 1) result from high rates of resource collection, which correspond to greater mission effectiveness.

### 4.2 Mission Efficiency

Mission efficiency aims to minimize usage of resources and time without compromising mission success.

In SAR missions, Jacoff et al. (2001a) and Jacoff et al. (2001b) used time to complete as a measure of mission efficiency, such that if the time stayed within set limits, then the team would receive extra points; otherwise, the team would lose points.

Steinfeld et al. (2006) introduced three efficiency metrics regarding time: mission completion time, operator time, and average time for extracting obstacles or average time to complete all sub-tasks. Chien et al. (2012) introduced the “event timeline” concept to also indicate the operator’s time, in a similar manner to operation loading (Jacoff et al., 2001a).

Following this discussion, it is evident that many metrics can be used for both effectiveness and efficiency. The difference lies in the definition of mission objectives and the scarcity of the resources. For instance, in an SAR mission, swarm power consumption can affect the effectiveness of the mission if the battery life of the robots is limited such that robots will not be able to progress after depleting the power. On the other hand, if power is a non-scarce resource, then power consumption becomes a metric of efficiency.

### 4.3 General Metrics for Mission Performance

We conclude this section with a set of common metrics that can be used in various missions for evaluating mission performance.

(1) Mission Effectiveness Metrics
  • Percentage of mission completion,
  • Total damage to the human-swarm system (e.g., number of robots damaged),
  • Mission constraints satisfaction, and
  • Number of undesired states (e.g., obstacles encountered).
(2) Mission Efficiency Metrics
  • Total completion time,
  • Time for completion of individual sub-tasks, and
  • Resource depletion (e.g., power consumption).

**FIGURE 2:**
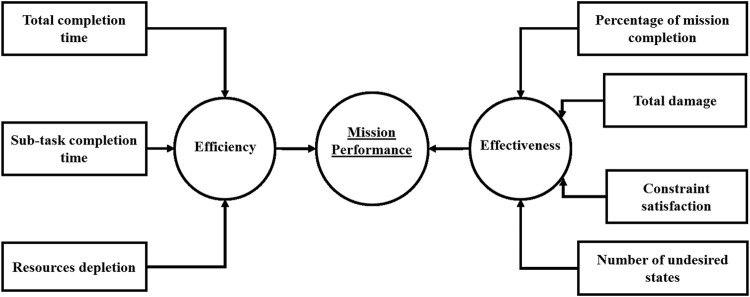
Examples of useful indicators for mission success.

These indicators introduced above are distilled to form the fusion sub-tree presented in Figure 2, where both measures of effectiveness and measures of efficiency form the two dimensions to measure mission performance.

## 5 Swarm Automation Level

The automation level of the swarm represents its capacity at a certain moment in time to complete its task without a need for human intervention. Finding effective metrics for the analysis of a swarm, as stated by Kolling et al. (2016), remains an open research problem. We will use the concepts of human dependence and neglect benevolence to combine and summarize the various metrics present in the literature.

Human dependence is a renaming of what Crandall et al. (2005) called neglect tolerance in their work, which is slightly different from the common definition of neglect tolerance in the literature (Olsen and Goodrich, 2003; Crandall and Cummings, 2007). To preserve consistency both within this paper and with the literature, we will use human dependence as a measure of the extent to which a robot is in immediate need of human intervention.

Human dependence corresponds to the composition of two different sub-metrics: neglect tolerance, which describes how the performance of the robot decreases while it is being neglected, and interaction efficiency, which describes how the performance of the robot increases when a human starts interacting with it after a period of neglect. Both of these measurements are correlated to the level of automation of the robot (e.g., a high automation robot will not suffer much from being neglected but could also experience reduced gains from human interaction), the complexity of the current situation, and previous history of interaction/neglect. The performance of a robot can then be described by the following equation:

$$P\left(\pi ,C,t\right)=\left\{\begin{array}{cc} P_{I}\left(\pi ,C,t_{\text{on}},T_{N}\right),\; & \text{if interacting}\\ P_{N}\left(\pi ,C,t_{\text{off}}\right),\; & \text{otherwise} \end{array}\right.$$ (2)

where *P* denotes performance, *P*_*I*_ denotes performance while the human is interacting with the robot, *P*_*N*_ denotes performance while the human is neglecting the robot, *π* denotes the current level of autonomy, *C* denotes the complexity of the situation, *t*_*on*_ and *t*_*off*_ denote the times since the start of the current interaction/neglect, and *T*_*N*_ denotes the time the robot had been neglected before the start of the current interaction.

Some useful metrics to estimate situation complexity *C* of the swarm can be found in Manning et al. (2015), and they are listed below:

• Cohesion: Evaluating the connectivity level of the swarm.
• Diffusion: Assessing the convergence and separation of swarm members.
• Centre of Gravity: Aiming to minimize the distance from the central point to other points in the spatial distribution of the swarm.
• Directional Accuracy: Measuring the accuracy between the swarm’s movement and the desired travelling path.
• Flock Thickness: Measuring the swarm’s density.
• Resource Depletion: Qualifying the irreversible consumption of limited resources by swarm members.
• Swarm Health: Evaluating the current status of the swarm.

In particular, swarm health is an important aspect for determining the difficulties faced by the swarm, and it can be decomposed by following the analysis by Harriott et al. (2014) into the following sub-components:

• Number of stragglers: studied by Parrish et al. (2002) as the number of fish of a school that have a distance of at least five body lengths from any other fish. This sub-component can reflect difficulties encountered by the swarm caused by obstacles in the environment or conflicting commands.
• Subgroup number and size: as explained by Navarro and Matía (2009), the number and size of subgroups can vary due to obstacles or as a way to perform the task more efficiently. In a swarm, subgroups can be identified and measured using clustering algorithms.
• Collision count: also studied by Parrish et al. (2002), this is the number of collisions between members of the swarm. If a collision avoidance system is in place, this could be the number of times this system had to intervene.

The other factor that is relevant to automation in an HSI system is neglect benevolence, which is a consequence of the fact that a swarm needs some time to stabilize after receiving an instruction before being ready to receive further instructions. In Nagavalli et al. (2014), this concept is formally defined and analyzed, leading to a complex algorithm for finding the optimal intervention time that requires computing the convergence time for the swarm with inputs given at different times. In practice, it may be possible to estimate the current value of neglect benevolence empirically by utilizing the time since the last human intervention and the factors that Walker et al. (2012) reported to be influenced by neglect benevolence: directional accuracy and cohesion. These two factors are both among those already used to compute the situation complexity.

**FIGURE 3:**
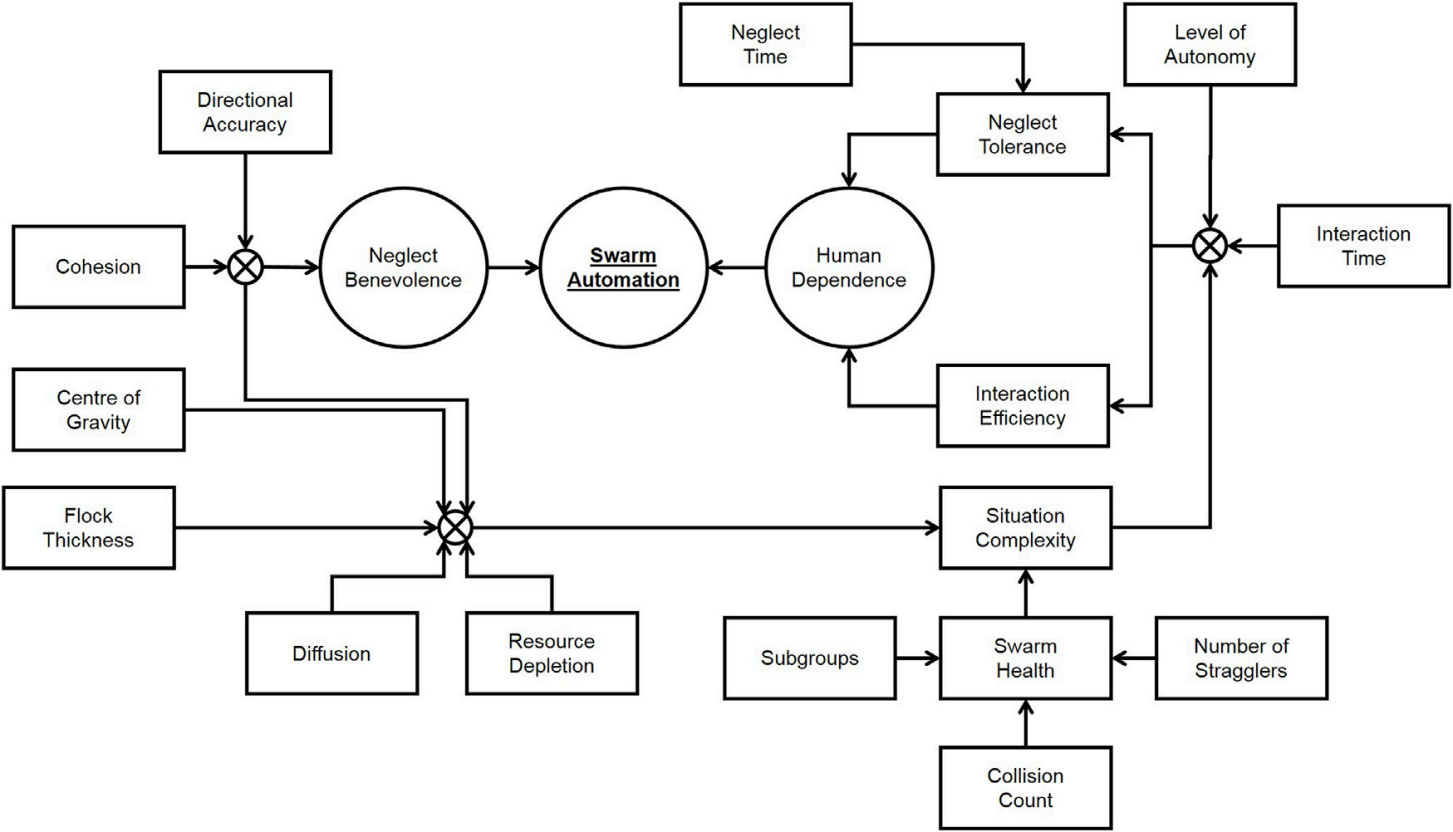
Metrics for swarm automation.

The concepts introduced in this section are composed in Figure 3, showing how they can be combined to build an estimation of the swarm automation level.

## 6 Interaction Indicators

The interaction between human and swarm refers to both the communication approach and the control methods that allow for exchange of their intent information and actions. It is natural that some of the factors that influence the swarm as mentioned above would also influence the interaction such as level of autonomy and neglect benevolence. A major challenge in HSI is the escalating complexity that could result from an increase in swarm size and task demands.

As the size of the swarm increases, the human must monitor and control an increasingly large group with massive numbers of interactions. For example, the human ability to control the swarm in a supervisory/manual control task would be severely limited by the cognitive capacity of human operators (Olsen Jr and Wood, 2004). Some modern techniques to control a swarm, on the other hand, use scalable control methods rather than controlling individual members and can maintain the workload at similar levels as the size of the swarm varies (Kolling et al., 2012; Pendleton and Goodrich, 2013). Nevertheless, the complexity of a problem may arise from the number and the structure of sub-tasks, which may require the division of a large swarm into smaller teams or the use of multiple swarms of heterogeneous entities. In these cases, introducing indicators for the effectiveness and efficiency of the interactions existing between a human operator and multiple high-level intelligent agents, each controlling a swarm, is important as both a form of detection tool for indication of when more or less automation is needed and as a diagnostic tool to understand the success (or otherwise) of the team.

There are three fundamental metric classes used in HSI introduced by Crandall and Cummings (2007): interaction efficiency, neglect efficiency, which reflects the efficiency of the agents performing the task without the attention of the human operator, and attention allocation efficiency, which captures the efficiency with which the human operator allocates his/her attention among multiple agents. These three metric classes are dependent on one another and also dependent on the level of autonomy that the influential AI and swarm component possess.

Interaction efficiency comprises different metrics discussed in the literature. The most popular metric is the interaction time, which is the amount of time needed for a human to manage one single entity in a multi-agent setting (Crandall et al., 2005). When dealing with multiple entities in the environment, this metric can be extended to Kerman (2013):

IEm=f(N(t))×interactiontime, (3)

where *IEm* is the interaction efficiency for multiple agents and *N*(*t*) is the number of agents the human interacts with at time *t*. The *f*(*N*(*t*)) term denotes a function describing the relationship between the number of agents and the time needed to manage the system (and the swarm controlled underneath). In the simplest case, this relationship might be linear with respect to the increase in the number of agents: *f*(*N*(*t*)) = *N*(*t*).

Neglect efficiency can be assessed by the neglect tolerance expressed by the amount of time an agent can be ignored before the error exceeds a threshold (Goodrich and Olsen, 2003). The neglect time has a direct relationship to the preservation of acceptable performance (Weitian Wang et al., 2018). Improving the neglect time is one goal of a successful HRI system, whereby the agent has enough capability to deal with the task. While we discussed neglect tolerance in the automation indicators, we still mention this metric here because it has an indirect impact on reducing the interaction effort.

Interaction effort provides information on how a particular interface design affects the overall effectiveness of the interaction. Interaction effort is defined both physically by the interaction time (Weitian Wang et al., 2018) and cognitively through the cognitive effort (Olsen and Goodrich, 2003) of sub-task choices, information requirement of the new situation after a choice, planning, and intent translation. When interacting with multiple agents, the interaction effort can be estimated indirectly via neglect tolerance and fan-out (the maximum number of agents the human is able to control effectively):

$$IEft=\frac{\text{neglect tolerance}}{\text{Fan}-\text{out}-1}$$ (4)

Attention allocation efficiency includes the situational awareness of the human with respect to the system and the environment (Zahabi et al., 2020). In a human-swarm interaction, this metrics consist of the switching time, and the time the human takes to decide to which agent in the swarm to switch his/her attention. When a human operates on a swarm with multiple substructures, the human must neglect some and prioritize his/her attention on controlling one agent to fulfil a corresponding sub-objective. Attention allocation efficiency captures this part of the interaction, where attention switching is occurring.

Intervention metrics are used to estimate the cognitive and physical efforts of a human when interacting with an autonomous agent. Interventions (Connolly et al., 2020; Chita-Tegmark and Scheutz, 2021) are unplanned interactions, as opposed to planned interactions in normal modes of operation. The intervention metrics include the average number of interventions over a time period, the time required for interventions, and the effectiveness of intervention (Scholtz et al., 2003; Senft et al., 2017). The efficiency of the interaction can also be evaluated through the ratio of intervention time to autonomy time (Yanco et al., 2004; Senft et al., 2017). For example, if the operator needs 1 min to give an instruction to agents and the agents then complete the task in 10 min, the ratio is 1:10.

This group of metrics has a strong connection to the level of autonomy that the swarm component possesses. In a shared control situation where there is a possibility for negotiation between human and automation, it is essential to identify extra measures such as the percentage of requests for assistance created by controlling agents (DelPreto et al., 2020; Kerzel et al., 2020), the percentage of requests for assistance created by the human operator, and the number of insignificant interventions by the human operator (Steinfeld et al., 2006).

Communication metrics capture those factors impacting the communication channels between the human and the swarm including latency and bandwidth, especially in the case of teleoperation or remote interaction with a large swarm. The problem of limited bandwidth was mentioned in McLurkin et al. (2006) in an attempt to design an effective interface for HSI, in which the centralized user interface is responsible for human command broadcasting, as well as integrating the information of the whole swarm in order to visualize them for the human operator. Kolling et al. (2016) reported a series of HSI experiments with different bandwidths. The findings supported the claim that the higher bandwidth offered larger capacity for multiple robots’ states acquisitions in a time step. An increase in latency caused degradation of interactions (Steinfeld et al., 2006; Walker et al., 2012). The problems mentioned above affect the effectiveness and the efficiency of the HSI because they impact the asynchrony of interaction among swarm members and delays in the bidirectional interactions. One solution for these problems may be a predictive display using swarm dynamics and bandwidth information.

**FIGURE 4:**
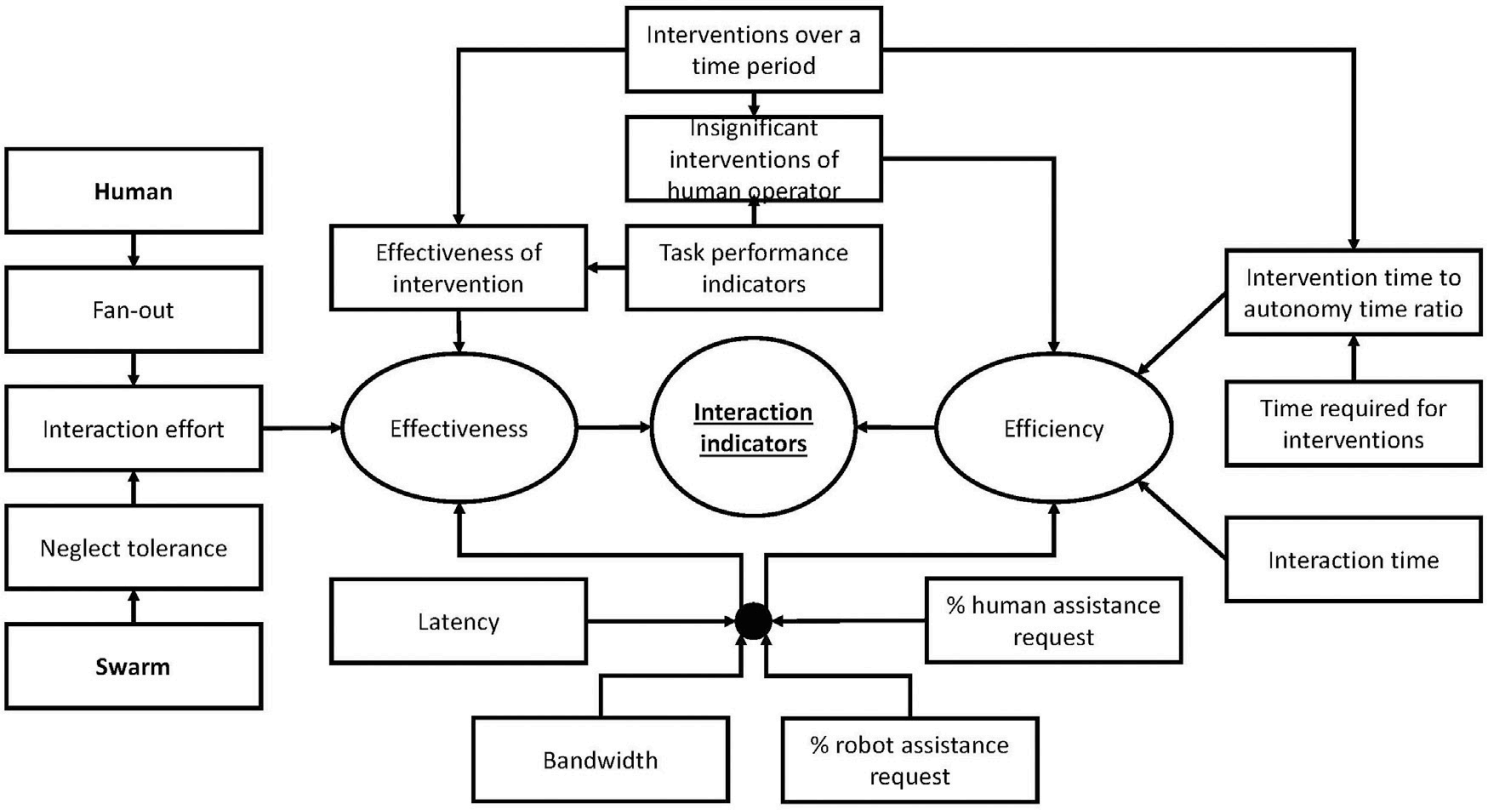
An example of a set of interaction indicators.

The relationship between the interaction metrics discussed above and the effectiveness and efficiency metrics of automation are summarized in Figure 4.

## 7 Human Cognitive States

Integrating human cognitive states into adaptive systems is a critical step towards effective and efficient HSI, for two reasons. First, real-time assessment of human cognitive states, such as cognitive workload, fatigue and attention, enables the system to adjust itself to maintain the human states within a safe envelope. This approach is particularly useful in scenarios where human mistakes caused by overload or underload and fatigue could potentially result in hazardous consequences. Second, human cognitive states can be translated into meaningful guidance for adaptation (e.g., swarm level of autonomy). It becomes pertinent to the adaptive HSI system to have a clear understanding of human cognitive states.

In HSI, the cognitive state of a user is a combination of variations among several interrelated constructs, including workload, attention, fatigue, and stress, to cite a few. However, to the best of our knowledge, there is currently no specific research on the cognitive state assessment in the context of HSI. Therefore, we discuss three types of measures for general human cognitive state assessment, followed by suggestions for HSI design.

Subjective measures are mainly in the form of questionnaires. They provide useful information on the self-assessment by a subject regarding the context of the question. This could range from pure collection of demographic data used to segment the subject population or identify confounding factors in the analysis to opinions on a matter of interest, including self-assessment of emotions. While this form of data gathering is unavoidable in certain contexts, its reliance on self-assessment by a user biases the results due to factors including personality, memory, social attitudes, and each user’s own understanding of the framing of the questions (Galy et al., 2018). Moreover, these measures cannot be collected continuously to provide the level of temporal resolution necessary for real-time usage.

Performance measures are based on the overt performances of users such as error rate, reaction speed, and task completion time. These measures are objective and can be continuously collected in real time, but only if they are carefully designed and integrated into the task (Mansikka et al., 2018). However, due to their task-dependent nature, these measures could disrupt users and be accompanied by low user acceptance.

Physiological measures include electroencephalography (EEG), event-related potential (ERP), Galvanic skin response (GSR), heart rate (HR), heart rate variability (HRV), electromyogram (EMG), eye movements (fixations, saccades, gaze, and blinks), pupil diameter, and respiration (Charles and Nixon, 2019). Each of these measures reflects a type of body response to cognitive manipulations. Table 1 summarizes the measures and a few of their functions.

**TABLE 1:** Physiological measures and a few of their functions.

Modalities	Functions (sensitive to)
EEG/ERP	Variations in mental workload (P300)
	Low/high-level perceptual and cognitive processes
	Alertness and task engagement
GSR	Arousal, stress and frustrations
HR/HRV	Cognitive demands, time restrictions, and uncertainty
	Attention, mental workload, and arousal
EMG	Motor preparation for movements and emotion
Eye movements	Task demands and fatigue
Respiration	Task demands and arousal

Among these modalities, EEG/ERP represents the most promising modality due to its high temporal resolution, accuracy, and sensitivity to dynamic changes of the cognitive states. The advantages of using physiological measures are as follows: they are objective metrics that do not rely on user perception, and they therefore support reliable measurements; multiple physiological measures can be integrated to provide a multidimensional profile of user states (Charles and Nixon, 2019); the collection of physiological data can be managed in an unobtrusive manner that does not directly interfere with user tasks; they are purely indicators that form implicit measures that are not based on an overt performance (Charles and Nixon, 2019); and last but not least, they are continuous data sources that naturally support real-time adaptation and dynamic control (Dehais et al., 2020).

Although the collection of physiological data often requires special signal acquisition devices (e.g., EEG recording devices), the rapid development of related technologies such as sensing technology (e.g., portable EEG cap with dry sensors) has made collecting data no longer a major concern. Moreover, practical issues related to the use of physiological signals in real-life scenarios have received increasing attention. For example, missing data or data disruption is a critical issue in practical applications, and machine learning models are proposed to address this data reconstruction problems (El-Fiqi et al., 2021). In addition, research on decoding and interpreting the collected physiological data towards meaningful cognitive indicators are developing fast, and the current results do indicate a promising future. For example, real-time EEG-based cognitive state assessment systems have been proposed and tested for user’s workload, fatigue, and attention in different application scenarios (Debie et al., 2019; Ladouce et al., 2019; Wu et al., 2020). A recent study demonstrates that it is feasible to establish profiles of the user motivations through EEG (Liu et al., 2021). In another study, the neural correlates of trust in human-autonomy interaction are identified, which allows for real-time assessment of human trust in automation (Min Wang et al., 2018). Furthermore, automatic recognition of the user identity is achieved with high recognition rate *via* EEG biometric systems (Wang et al., 2019; Wang et al., 2020). Integrating these cognitive state indicators for workload, fatigue, attention, and potentially motivation, along with the user identity will generate a dynamic user profile to support adaptive control.

In summary, in an HSI setting, a user needs to at least monitor the cloud where the swarm is embedded with significant attention load. It seems pertinent to rely on physiological measures when designing cognitive indicators for adaptive HSI systems. Each measure has its own bias and is unlikely to be useful on its own in general situations. This situation calls for the need to use multiple modalities (Debie et al., 2019) and engineer fusion techniques to design appropriate informative pictures of human cognitive states in real time.

## 8 Mission Complexity

Mission complexity indicates the overall level of effort needed by both humans and swarm to perform the mission. In a teleoperation scenario, mission complexity is in the hands of a human alone. In a shared control scenario, mission complexity is distributed between the humans and the swarm. In essence, it is the responsibility of the adaptive agent to decide how to distribute the functions during function reallocation. In this section, and without loss of generality, we examine this component of mission complexity that is assigned to the human; thus, while the previous section examined indicators of workload, this section examines causes of workload, that is, mission complexity. At zero-level autonomy, the component assigned to a human is simply the overall mission complexity of the HSI. For a fully autonomous system that does not require any human oversight or interaction, the mission complexity component assigned to the human is zero. Between these two extremes, the component assigned to the human, and therefore influencing human workload, is general enough to cover the overall mission complexity concept.

Mission complexity influences the amount of mental workload that a mission will potentially require from a human. Because human workload can negatively hinder the success of a mission that relies on collaboration between the human and the swarm, the continuous monitoring and adaptation of mission complexity becomes crucial. Although human workload can be measured directly using psycho-physiological techniques, as discussed in Section 7, mission complexity is distinct in two ways. First, mission complexity considers only workload associated with the mission, such that if a portion of the workload experienced by a human is related to some factors external to the mission, this portion will not be accounted for by mission complexity. Second, complexity metrics play a diagnostic role by identifying how each task contributes to the overall mission-related workload. This is particularly important to the adaptive agent as it provides the required information on how a certain adaptation could potentially result in a desirable workload level. In this section, we first discuss different factors of complexity, and then, we show how these factors form the three components of mission complexity.

### 8.1 Factors of Complexity

Both objective and subjective factors can impact the mission complexity for, and therefore the performance of, a human (Maynard and Hakel, 1997). Objective factors can stem from the task structure, the interface, or the environment, while subjective factors stem from human experience, skills, and self-confidence. The main focus of this section is the objective factors of mission complexity. We will divide these factors into three groups, depending on whether they are caused by the swarm, the interface, or the structure of the mission.

#### 8.1.1 Swarm Characteristics

Within a team setting, properties of the teammates—the swarm—can considerably impact mission complexity. Two basic swarm characteristics have been identified in the literature as affecting human mental workload: the level of autonomy and size. The level of autonomy of a swarm was shown to be an important source of complexity. Ruff et al. (2002) studied the workload associated with different levels of autonomy while navigating a group of four UAVs. They found that manual control resulted in the highest level of workload. Riley and Endsley (2004) found the same result in robot-assisted search and rescue missions. Mi and Yang (2013) argued that these results also generalize to swarm operation. However, increasing the level of autonomy as in semi-autonomous swarms does not lead to the omission of workload. In principle, this setting requires considerable cognitive resources as the human has to understand a plethora of information arriving from the swarm (Cummings, 2004) in order to maintain a high level of situational awareness (Endsley, 2017).

The size of the swarm can also result in increasing workload requirements, particularly in manual control settings (Ruff et al., 2002). Scalable control methods, rather than controlling individual members, reduce the sensitivity of workload to swarm size. For instance, Kolling et al. (2012) proposed two methods for controlling the swarm in a foraging task: selection and beacon. They showed that the number of human instructions did not change significantly across different swarm sizes. Pendleton and Goodrich (2013) also used three control methods in a foraging task: leader, predator, or stakeholder. They found that using these control methods does not result in a significant change in the workload across different swarm sizes. It is worth mentioning that in real environments, scalable control methods would reduce but not eliminate the effect of swarm size on mission complexity. Performance drops encountered by individual robots or sub-swarms would require human intervention (Wang and Wang, 2017). The frequency of such events is impacted by swarm size leading to increased complexity.

#### 8.1.2 The Interface

The interface between the team players, with the human on one side and the swarm on the other side, can contribute to mission difficulty. Interface complexity can stem from the activated swarm control method, the information presented, and the display technology used. Irrespective of the level of autonomy of the swarm and the number of swarm members, the active control method can affect the complexity of the interface. For example, Pendleton and Goodrich (2013) found that both control by a leader and by a stakeholder result in lower workload than control by a predator.

Information presentation decisions with respect to the amount and level of information presented are another source of interface complexity. The amount of information affects cognitive load such that too little information results in increasing uncertainty and leads humans to integrate information from other sources such as their own assumptions, which could increase cognitive load (Van Der Land et al., 2013). Excessive information, on the other hand, makes the human overwhelmed with a large quantity of data that may exceed their cognitive capacity (Roundtree et al., 2019). The impact of the level of information was also examined by previous works. Riley et al. (2010) argued that low-level information negatively impacts operators’ cognitive load, as they must process it to build higher levels of SA (Riley and Strater, 2006). In contrast, swarm-level information enables the human to make sense of swarm behaviors leading to effective collaboration (Hussein et al., 2020a). Finally, communication issues impose limits on the amount and speed of information exchange between the human and the swarm. These issues can lead to increased complexity in scenarios where human actions might be based on outdated or incomplete information about the swarm (Hepworth et al., 2021).

#### 8.1.3 Task Structure

The structure of the mission and how it is executed is a third source of complexity. For instance, the existence of tasks that are executed concurrently adds to the human workload by increasing the information load (Liu and Li, 2012). Chen and Barnes (2014) argued that switching between tasks can increase workload due to the possible interference between task-related information. This interference increases if tasks are similar with respect to stimuli, processing stages, or required responses (Harriott, 2015). It has been found that a human may require upto 7 s to recover task-related SA when switching between tasks (Chen and Barnes, 2014). Problem-space factors can also impact the complexity of a task. For example, obstacle density affects the complexity of navigation tasks (El-Fiqi et al., 2020), whereas conflicting evidence affects the complexity of a decision-making task (Hussein et al., 2020b).

### 8.2 Components of Complexity

Throughout the mission, a human can be involved in three types of activities: action execution, SA formation, or SA restoration. These classes of activities will be considered as the main components that constitute mission complexity.

#### 8.2.1 Action Execution

The number of actions a human needs to perform depends on the level of autonomy of the swarm, the control method, and the size of the swarm. At low levels of autonomy, the human has to execute a variety of actions at both low and high levels, e.g., tele-operated navigation and target identification, respectively. As the level of autonomy increases, a human becomes mainly responsible for mission-level tasks, while the swarm takes over control of low-level tasks. The scalability of the control method used will determine how frequently a certain type of action needs to be performed in relation to the number of swarm members. For instance, the task of identifying regions of interest in the environment will be performed a fixed number of times. However, identification of targets found by each swarm member will be repeated a number of times proportional to the number of swarm members. Thus, the total number of actions a human needs to perform will be the product of types of different actions, multiplied by the frequency of executing each type of action.

#### 8.2.2 Situational Awareness Formation

While supervising the swarm within a mission, the human needs to attend to and integrate the incoming information to acquire the SA. The complexity of SA formation comes from the amount, level, and quality of the information presented. As the amount of information increases, the human will need to exert more effort to perceive these pieces of information. For example, presenting the health level of each swarm member can be more mentally demanding than presenting the average and standard deviation of the health of the swarm as a whole. Moreover, if the information is presented in a primitive form, the human needs to integrate it with previous pieces of information to understand the situation. Information quality issues will require additional effort from the human to estimate and account for potential uncertainty in the data.

#### 8.2.3 Situational Awareness Restoration

As the number of concurrent tasks and interruptions increases, the human will more often need to leave a certain task for a while to execute other tasks or respond to active interruptions. When switching back to the old task, a human needs to exert effort to restore the SA of the task and to catch up with any updates that took place while executing other tasks. The difficulty of restoring the SA will increase as the similarity between the tasks increases due to possible interference. Thus, the complexity of restoring SA is a function of both the number of concurrent tasks and the similarity between tasks.

**FIGURE 5:**
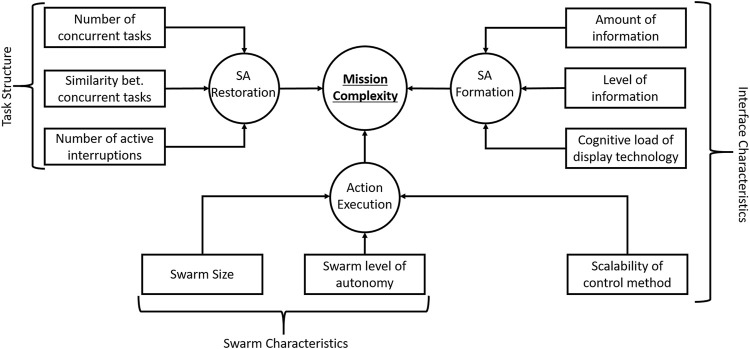
Components of mission complexity.

The concepts introduced in this section are composed in Figure 5, showing how they can be combined to build an estimation of the mission complexity.

## 9 All in One: The Mission-Interaction-Complexity-Automation-Human Framework

**FIGURE 6:**
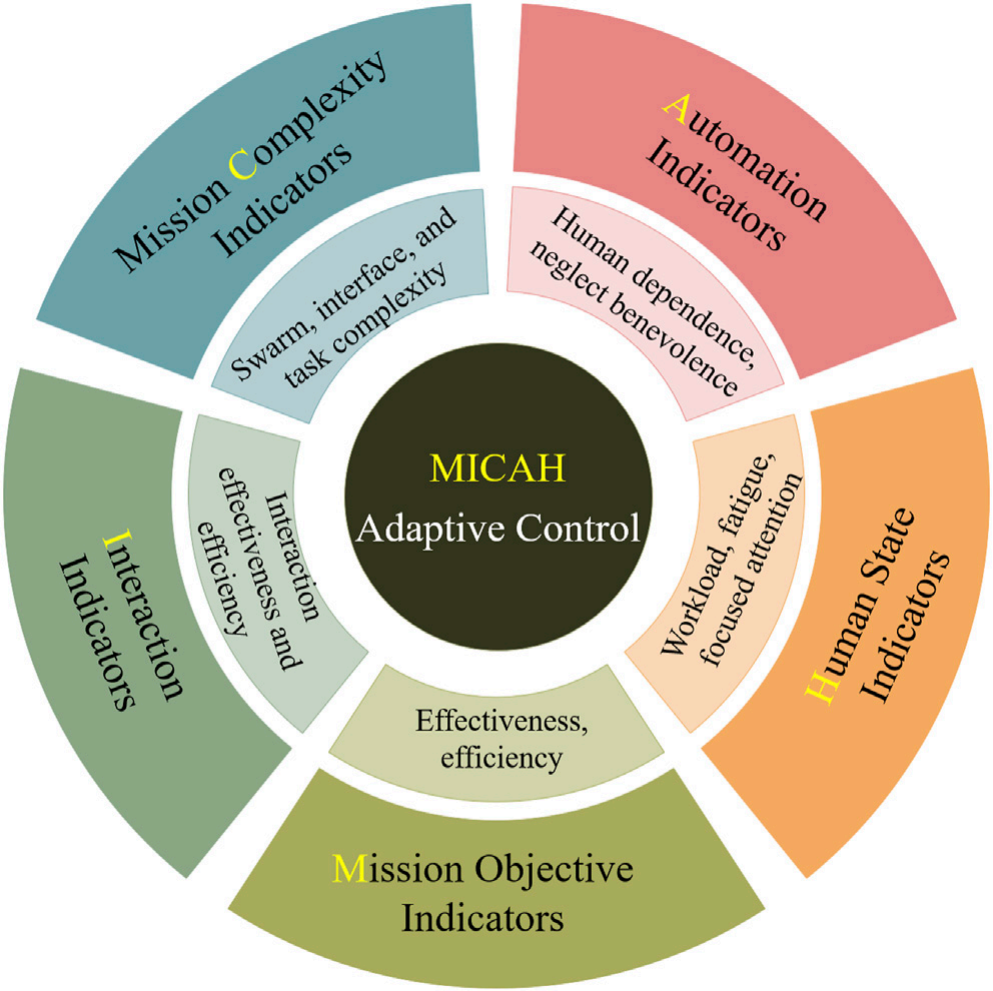
MICAH: categories of indicators used for adaptation in HST.

In Sections 4–8, we presented the five types of indicators that need to be brought together for the adaptive AI agent to manage the interaction in HSI systems. Figures 2–5 summarize how such indicators can be calculated from the raw data. The context the HSI system is operating within may necessitate replacing and/or augmenting the particular metrics that we discussed. Nevertheless, all five types are necessary, and the inter-dependencies among them need to be clearly defined to ensure an effective design of the adaptive AI agent. We named this framework with the acronym MICAH, and a visual summary of it is presented in Figure 6.

The five types of indicators can be summarized in the following way, with the letter contributing to the abbreviation MICAH underlined:

• Mission performance is the ultimate objective of the system and should never be disregarded. It can be measured by effectiveness and efficiency metrics.
• Interaction provides a quantification of the productivity of the interaction between the human and the swarm. Monitoring interaction indicators helps to evaluate the current interaction mode.
• Mission Complexity provides diagnostic information on the objective factors that contribute to the workload imposed on the human, hence suggesting avenues for reducing the workload if necessary.
• Automation level analyses the performance of the swarm and its need for human intervention, which are fundamental inputs to correctly set the level of autonomy.
• Human cognitive states assess the mental conditions of the human, determining, for example, if they are overloaded or underloaded to allow the system to adapt accordingly.

The following is a walk through the SAR scenario described in Section 3.1 to demonstrate the overall operation of the framework. Consider the current level of autonomy is set such that the human is responsible for prioritizing search regions and identifying victims based on images captured by the UGVs, while path planning is assigned to the swarm. Table 2 lists the specific indicators selected for each component of MICAH in this scenario. In the beginning of the mission, the mission performance is satisfactory and the human workload level is within an acceptable range, i.e., high *P*_*collection*_ and medium *L*_*workload*_. These assessments indicate that the current HSI setting is appropriate and that no adaptation is required.

**TABLE 2:** Selected indicators for the SAR scenario.

Categories	Indicators	Notations
Mission performance	Victim collection rate	*P*_*collection*_
	Time for completion of individual sub-tasks	*T*_*task*_
Interaction	Change in performance after interventions	*δ*_*interven*_
Complexity	Number of assistance requests received from the swarm	*N*_*request*_
Swarm automation level	Number of UGV stragglers	*N*_*straggler*_
	Collision count	*N*_*collision*_
Human cognitive states	Cognitive workload level	*L*_*workload*_

Then, the swarm starts navigating through a narrow corridor with obstacles scattered along it. The mission effectiveness and efficiency start declining (i.e., *P*_*collection*_ is getting low and *T*_*task*_ is getting high) due to the difficulty of navigating through obstacles. This decline in mission performance activates the adaptive control module, but does not specify the cause of the problem (the human or the swarm), and hence the desirable direction of adaptation. Swarm automation indicators provide more of the required information, as they indicate poor swarm performance due to the increase in collision counts *N*_*collision*_ and number of stragglers *N*_*straggler*_. These changes are accompanied with an increase in the mission complexity indicator *N*_*request*_. Thus, the adaptive control logic switches path planning from the swarm to the human. A lower-level control method is activated, which allows for both controlling the swarm as a whole and controlling individual UGVs if required. The interface changes accordingly to present low-level information.

The efficacy of the intervention of the human is monitored. There is a positive change in performance after human intervention (i.e., *δ*_*interven*_ is high) which shows that the human is able to support the swarm operation. The swarm starts to cross to the other side of the narrow corridor into a space with more degrees of freedom where *N*_*straggler*_ and *N*_*collision*_ start decreasing. After the successful navigation through the narrow corridor, the human workload level *L*_*workload*_ exceeds the safety level. The adaptive control module is activated again, and a decision is made to switch the path planning responsibility back to the swarm. The monitoring continues till the end of the scenario so that adaptation can be activated as needed.

Regarding the adaptive agent, there are various techniques that can be used to implement its functionality. For instance, the agent can be built as a set of IF-THEN rules by relying entirely on domain experts to identify when and how adaptation should be performed. In many situations, there could be some vagueness in the language used for defining these rules. Fuzzy logic can be useful in these situations as it allows for some vagueness in defining the rules and tolerates uncertainty in the values of the metrics (Mittal et al., 2020). However, it can be a challenging task for a domain expert to generate these sets of rules in the first place, as this process requires thinking into how to make an adaptation decision based on all the different combinations of values for the five indicators.

Machine-learning techniques can leverage training data to assist with building an adaptive logic agent. The data can be collected by simulations in advance to compile a set of examples of how values of the MICAH metrics can be mapped into an appropriate level of autonomy. These examples can be then used to train a model for adaptive logic in a supervised learning setting. Alternatively, the adaptive logic agent can be trained using trial and error in a reinforcement learning (RL) setting. For instance, the inputs to the RL adaptive agent would consist of both the metrics used for the five indicators and the current task assignment (i.e., the level of autonomy). Meanwhile, the output of the RL agent would be a set of discrete actions representing the task assignment for the next time frame. The RL agent would be requested to produce an action each *τ* minutes or as triggered by emergency situations. The RL agent can be employed within a series of (possibly simulated) missions to learn a useful adaptation policy via interaction with the environment. By properly designing the reward function, the RL agent can learn policies for various objectives (e.g, maximizing the performance or balancing the load between the human operator and the swarm). To bootstrap the model parameters, inverse reinforcement learning can be employed to learn from demonstrations by a domain expert (Arora and Doshi, 2021; Nguyen et al., 2021).

## 10 Conclusion and Future Work

In this paper, we proposed a framework that extends existing concepts of adaptive systems to fit swarm systems in order to achieve effective and efficient HSI. We brought together literature from different fields including HRI, HSI, task complexity, and psycho-physiological techniques to identify and discuss classes of indicators that convey complementary information that is significant for effective adaptive autonomy.

The adaptive AI agent includes two phases: monitoring and assessment; and adaptation. We focused this paper on the first phase. The mapping from state assessment to a certain adaptation is by no means trivial and will be the focus of our future extension of this work, wherein we will aim at designing the technical details of the adaptation systems based on the proposed framework. The use of human experiments will represent an invaluable source for learning and evaluating adaptation strategies. However, to obtain sufficient data that span the state space, human experiments would be very costly in terms of their financial and temporal needs. Hence, the use of models that closely capture the relevant system aspects can be quite effective in this scenario to obtain the required data (Hussein and Abbass, 2018). In this way, different adaptation strategies can be explored and tested in the simulation platform using techniques like reinforcement learning. This approach would enable the mapping from mission states to adaptation actions based on the learned (long-term) rewards associated with these actions.

To validate the effectiveness of this framework, it will be instantiated and evaluated in a dynamic environment similar to the scenario described in Section 3.1. Depending on the specifics of the test scenario, one or more metrics will be used from each class of indicators to inform the adaptation decision. To evaluate the monitoring and assessment phase, it will be necessary to investigate the contribution of each class of indicators to the dynamic adaptation. Hence, a simple rule-based adaptation manager will be implemented in which each class of indicators will be added incrementally, starting with no indicators (base-line system with static task allocation) and ending with an adaptation manager that uses all five classes of indicator. The corresponding change in performance can then be used to determine the added value of each class of indicators or lack thereof. The second phase, adaptation, can similarly be tested by comparing the performance of the AI-based adaptation manager to a rule-based adaptation manager, given the same input data.

It is worth mentioning that obtaining data for the five indicators may not always be a straightforward process. For example, to assess the swarm automation level, it is implicitly assumed that the adaptive AI agent can observe the state of the swarm and its operational environment. In remote environments, the quality of these observations can drop significantly due to, for example, limited bandwidth. In these situations, statistical summaries of information about the state of the swarm can be calculated at the swarm side before sending it (Nunnally et al., 2012). This approach may require efficient algorithms for evaluating the swarm state or the environmental features in question and electing a swarm member to communicate the information back to the adaptive AI agent.

The accurate interpretation of human cognitive states based on the psychophysiological data remains a challenge due to the difficulties in discriminating mental states (e.g., attention, workload, and fatigue) in real settings (Lohani et al., 2019). However, continuous research promises increasing utility of the physiological measurements in the near-real-time estimation of the cognitive states.

When one data source becomes unavailable, reliance on multi-modality becomes a necessity (Lahat et al., 2015; Debie et al., 2019). Moreover, multi-modal fusion techniques offer more robust indicators and resilience to noise, data loss, and missing information (Wagner et al., 2011). By learning the relationships between data from the five indicators, techniques to infer missing data from the existing data can be used (Srivastava and Salakhutdinov, 2012).

Last, but not least, it is anticipated that the adaptive AI agent will have a learning component that dynamically learns from the data provided by the MICAH framework. For example, changes in the EEG data of a human cannot always be interpreted without context and normally differ from one subject to another. The adaptive AI agent may use association rule mining in continuous domains (Wang et al., 2016) to associate changes in task and mission complexity with changes in particular user EEG indicators. In addition, as discussed in Section 9, the functionality of the adaptive control agent can be implemented through multiple ways, including a set of rules, fuzzy logic, reinforcement learning and other machine learning techniques. Among these methods, rule-based or decision tree-based methods have advantages in improving the transparency and explainability of decisions made by the adaptive control agent (Dorigo et al., 2021). Methods based on deep learning algorithms, on the other hand, are able to make full use of the available data and have the potential in providing more effective and robust representations for adaptive control (Benosman, 2018). In our future study, we will consider both directions in the implementation and validation of the MICAH framework in practical applications.

## Data Availability

The original contributions presented in the study are included in the article/supplementary material, further inquiries can be directed to the corresponding author.
